# Corneal Collagen Crosslinking Treatment in a Case with Pneumococcal Keratitis

**DOI:** 10.4274/tjo.98470

**Published:** 2017-06-01

**Authors:** Ayşe Bozkurt Oflaz, Banu Bozkurt, Ümit Kamış, Bengü Ekinci Köktekir

**Affiliations:** 1 Selçuk University Faculty of Medicine, Department of Ophthalmology, Konya, Turkey

**Keywords:** Bacterial keratitis, corneal collagen crosslinking, ultraviolet-A/riboflavin

## Abstract

Bacterial keratitis is a serious ocular infectious disease that can threaten vision. The disease generally progresses rapidly and can lead to corneal scar, stromal abscess formation, perforation, and dissemination to adjacent tissues if not treated properly. Recent studies showed that corneal collagen crosslinking (CCC) using ultraviolet-A/riboflavin is effective in the treatment of bacterial keratitis refractory to topical antibiotic treatment. In addition to being bactericidal, CCC also decreases risk of perforation by strengthening the corneal collagen structure. Herein, we report a male patient with Streptococcus pneumonia keratitis 6 months after a keratoplasty procedure, which did not respond to fortified topical antibiotic therapy and was treated successfully with riboflavin/ultraviolet-A CCC. His pain decreased remarkably in a few days. The corneal epithelial defect healed and infiltration regressed within 2 weeks after CCC. His vision improved significantly from hand movement to 20/400. CCC might be used as adjuvant treatment in bacterial keratitis refractory to medical treatment.

## INTRODUCTION

Bacterial keratitis is the leading sight-threatening ocular infection. The disease generally progresses rapidly, and may lead to corneal scar, stromal abscess, perforation, and dissemination to adjacent tissues if not treated properly.^1^ Bacterial keratitis usually occurs in the presence of risk factors leading to disruption of the ocular surface immune mechanisms. Contact lens use; dry eye disease; eyelid disorders such as entropion, ectropion, and lagophthalmos; ocular surgery; and long-term corticosteroid use are among the risk factors.^2,3^ Topical application of broad-spectrum, bactericidal antibiotic agents is used to treat bacterial keratitis.^4^

Corneal collagen crosslinking (CXL) is an extremely effective therapy shown to arrest the progression of keratoconus, pellucid marginal degeneration, and post-LASIK corneal ectasia, and has become widely used worldwide over the last decade.^5,6,7^ In the procedure, ultraviolet-A (UV-A) irradiation causes riboflavin to form triplets and release reactive oxygen species such as singlet oxygen and superoxide, which form new covalent bonds between the amino acids of adjacent collagen fibrils (photopolymerization). This polymerization increases the rigidity of corneal collagen and improves resistance to keratectasia. Furthermore, the application of UV-A and riboflavin has been shown to inactivate certain viruses, bacteria, fungi, and parasites in several in vitro studies.^8,9,10^ Clinical studies have also demonstrated that CXL is safe and effective in the management of corneal infections refractory to medical treatment.^11,12,13,14,15^ CXL also reduces the risk of perforation by strengthening the cornea. Despite the widespread use of CXL in the treatment of keratoconus in Turkey, were unable to find any Turkish publications regarding its use in cases of infectious keratitis. In this report, we present the case of a keratoplasty patient with *Streptococcus pneumonia* keratitis refractory to topical antibiotic therapy who showed rapid clinical improvement after CXL, and discuss the case in the context of the literature.

## CASE REPORT

**Figure 1:**
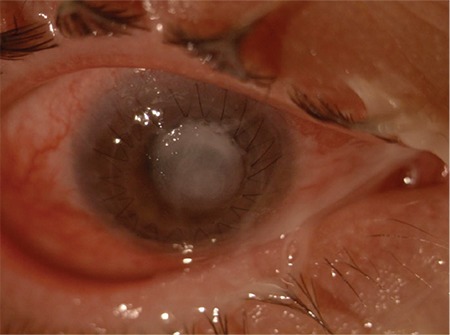
Infiltration in the center of the corneal graft, conjunctival hyperemia, and purulent secretion

**Figure 2:**
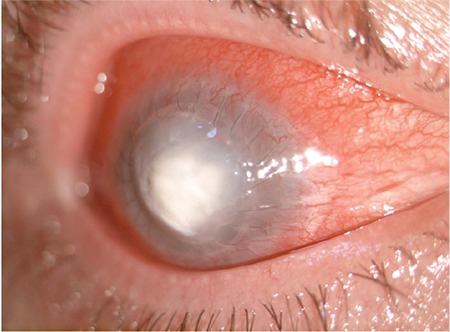
Corneal infiltration progressed and a large epithelial defect and peripheral corneal neovascularization developed despite topical fortified antibiotic therapy

A 32-year-old male patient presented in October 2014 with complaints of pain, blurred vision, and photophobia in the right eye for approximately 1 week. In his medical history, he reported being followed at another center for keratoconus and undergoing keratoplasty 7 years earlier in his left eye and 6 months earlier in his right eye. He had been using moxifloxacin (Vigamox^®^, Alcon) every 3 hours for about 1 week. At presentation, his visual acuity was hand motions in the right eye, and his best corrected visual acuity in the left eye was 8/20. Slit-lamp examination of the right eye revealed conjunctival hyperemia, purulent secretions and a keratitis focus about 5x4 mm in size located centrally over the corneal graft, epithelial defect, and keratoplasty sutures (Figure 1). The patient was recommended for admission with a prediagnosis of infectious keratitis; swabs from over the infiltration and its margins were sent to microbiology for Gram staining and culture. There were no filamentous extensions, satellite lesions, or ring ulcers suggestive of fungal keratitis. We also detected no hyphae-like structures suggesting fungal infection on confocal microscopy. Moxifloxacin was discontinued and treatment was initiated with topical fortified vancomycin 50 mg/mL (Vancotek^®^, Koçak Pharmaceuticals) and ceftazidime 100 mg/0.5 mL (Zıdım^®^, Tum Ekip), alternating between them hourly and replacing the drops every 2 days. Gram staining revealed no bacteria in the swab specimens, but *Streptococcus pneumoniae* grew in culture toward the end of the second week. Antibiogram showed the isolate was sensitive to vancomycin and ceftazidime, so the same treatment regimen was continued. Considering the lack of substantial improvement in the patient’s symptoms and the possibility of Candida superinfection due to long-term topical steroid use after keratoplasty, treatment was supplemented with topical amphotericin B 0.5 mg/mL (Fungizone^®^, Bristol-Meyers Squibb) every 2 hours and topical vancomycin/ceftazidime was reduced to every 3 hours. After 1 month of medical therapy, we observed that the corneal infiltration had deepened and the epithelial defect had not closed (Figure 2). Therefore, we decided to perform CXL with UV-A and riboflavin. The patient was fully informed about the procedure and provided informed consent before the procedure.

In operating room conditions under topical anesthesia, the eye and surrounding area were cleaned with 5% povidone iodine and a sterile covering was placed. The damaged epithelial tissue over the infiltration was debrided using a blunt spatula and sent to microbiology. A riboflavin solution (1% isotonic M) was applied to the cornea at 3-minute intervals for a total of 30 minutes. An area of the cornea about 7 mm in diameter was exposed to 365-370 nm UV-A from a distance of 4-5 cm for 30 minutes at 3 mW/cm^2^. During this time, riboflavin solution and a lubricating agent (Tears Naturale Free^®^, Alcon) were applied every 4 minutes.

**Figure 3:**
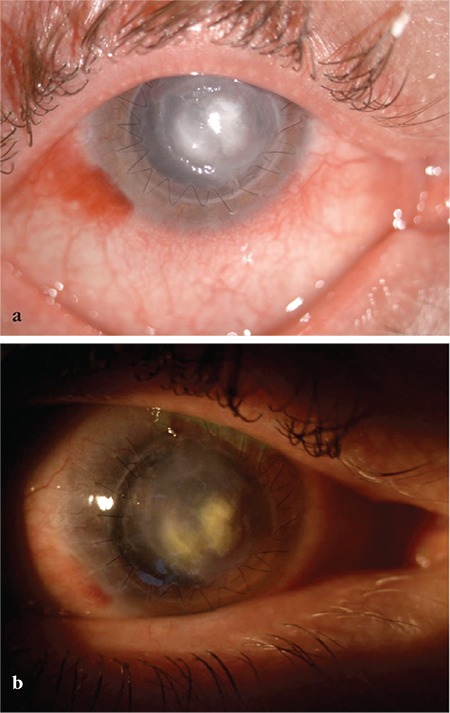
Examination at 1 day (a) and 1 week (b) after corneal collagen crosslinking revealed that the corneal infiltration diminished in size and split into two foci, the surrounding corneal tissue regained transparency, and the epithelial defect became smaller

**Figure 4:**
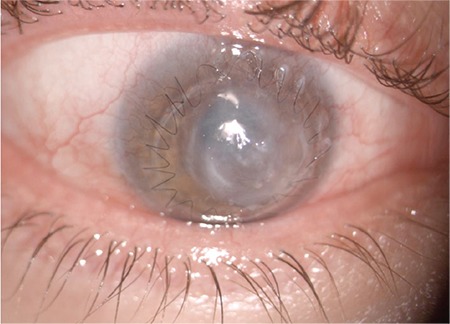
At 1 month after the corneal collagen crosslinking procedure, the corneal infiltrate and epithelial defect had resolved and there was slack in the corneal sutures

**Figure 5:**
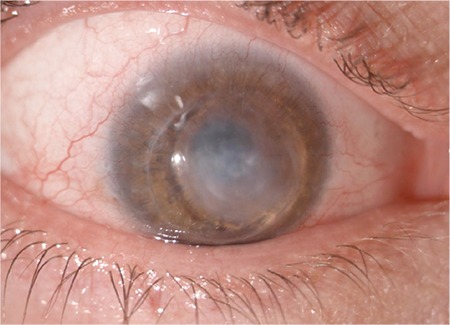
At 6 weeks after the corneal collagen crosslinking procedure, the corneal sutures were removed, peripheral corneal neovascularization had regressed, and corneal opacity was reduced

There was a substantial reduction in the patient’s photophobia and pain the day after CXL treatment. In subsequent examinations, the corneal infiltration became smaller and split into two foci, the surrounding corneal tissue regained transparency, and the epithelial defect also became smaller (Figure 3). The dosage of topical antibiotics was reduced. On examination 1 month after the CXL procedure, we observed slackening of the keratoplasty sutures and corneal vascularization (Figure 4). The keratoplasty sutures were removed and subconjunctival anti-vascular endothelial growth factor (anti-VEGF) (bevacizumab, Avastin^®^, Genentech) was administered. Preservative-free artificial tears (Tears Naturale Free^®^, Alcon) 5 times daily and fluorometholone (Flarex^®^, Alcon) 3 times daily were initiated. At 6 weeks after the CXL procedure, the peripheral corneal neovascularization had regressed, corneal opacity was reduced, and visual acuity was 20/400 (Figure 5).

## DISCUSSION

Riboflavin is able to pass through the lipid cellular membrane and be incorporated into nucleic acid chains. When activated by UV-A light, riboflavin releases reactive oxygen species that oxidize nucleic acids, thereby damaging the DNA and RNA of pathogens.^9,10^ Pathogenic microorganisms produce certain enzymes that can destroy collagen and lead to corneal erosion and perforation. By eliminating pathogenic microorganisms and strengthening the collagen structure, CXL is able to halt the enzymatic destruction of the corneal stroma and reduce the risk of perforation. Previous studies have demonstrated the antibacterial effect of the combination of riboflavin and UV-A against multidrug-resistant *Pseudomonas aeruginosa, Staphylococcus aureus, S. epidermidis*, methicillin-resistant *S. aureus, S. pneumoniae*, and *Escherichia coli*.^9,12,13^

Iseli et al.^11^ reported regression in the infectious keratitis foci of 5 patients being treated with topical and systemic antibiotic therapy after CXL treatment. In all patients, corneal ectasia was arrested and emergent corneal transplantation was not required. Makdoumi et al.^14^ showed that CXL with riboflavin and UV-A could be used in combination with antibiotic therapy in a study of 7 eyes with severe keratitis. In another study, Makdoumi et al.^15^ performed CXL as primary therapy in 16 cases of microbial keratitis. Other than 2 cases that required antibiotic therapy, all eyes showed epithelial healing and reduced inflammation. Price et al.*16* reported that CXL performed in addition to medical therapy successfully controlled infection in 34 of 40 eyes with keratitis (24 bacterial keratitis, 7 fungal keratitis, 2 protozoal keratitis, 1 viral keratitis, 6 unknown; 18% of eyes had keratoplasty). Treatment with CXL is especially effective with bacterial keratitis and with less deep infections.

Our patient developed severe *S. pneumoniae* keratitis in the eye that underwent keratoplasty 6 months earlier, and did not respond well to treatment despite antibiogram which indicated sensitivity to moxifloxacin, vancomycin, and ceftazidime. Because he was treated on an inpatient basis and his antibiotics were replaced every 2 days, we do not believe there were any issues concerning treatment compliance or drug stability. The lack of treatment response may be attributable to microbial in vivo drug resistance, inadequate penetration of the drug into the cornea, toxicity and impaired healing due to long-term drug use, or the persistence of inflammation and tissue damage despite controlled infection. We performed CXL after 1 month of treatment in order to eliminate resistant microorganisms and reduce the risk of perforation by strengthening the cornea. Immediately following the procedure, the patient reported a significant reduction in pain; within a few days, the epithelial defect began to heal rapidly and the corneal infiltration became smaller and more superficial, healing with mild scarring.

Although there are studies in the literature supporting the use of CXL therapy, other reports argue that the procedure should not be routinely used in the management of infectious keratitis and emphasize that it may have a toxic effect in the diseased cornea, particularly on the endothelium. Kashiwabuchi et al.^17^ found CXL ineffective both in vitro and in vivo against *Acanthamoeba* trophozoites. Galperin et al.^18^ showed that riboflavin-CXL reduced the intensity and severity of infection but did not provide adequate healing in a experimental rabbit model of *Fusarium* keratitis. In addition, as UV-A can induce viral replication, it should not be used in cases of herpes simplex keratitis.^19,20^ Demirci and Ozdamar^21^ found that CXL was effective in patients with *Acanthamoeba* keratitis refractory to medical therapy.

In summary, CXL with UV-A and riboflavin may be effective in cases of bacterial keratitis refractory to medical therapy. Additional studies are necessary to determine the efficacy and reliability of CXL in infections caused by various microorganisms.
